# Evidence from Clinical Studies Related to Dermatologic Surgeries for Skin Cancer

**DOI:** 10.3390/cancers14153835

**Published:** 2022-08-08

**Authors:** Shoichiro Ishizuki, Yoshiyuki Nakamura

**Affiliations:** Department of Dermatology, Faculty of Medicine, University of Tsukuba, Tsukuba 305-8575, Ibaraki, Japan

**Keywords:** skin cancer, surgical margin, lymph node dissection, skin graft

## Abstract

**Simple Summary:**

Although significant progress in pharmacotherapy for skin cancer has been made in the past several years, surgical removal of primary skin cancer is still the first choice of treatment unless distant metastases are evident. In the surgical treatment of primary skin tumors, the surgical margin is critical not only for reducing the possibility of tumor recurrence but also for minimizing the cosmetic and functional complications associated with wide local excision. In contrast, dermatologic surgeries including lymph node dissection and skin graft can cause various complications, and these complications are frequently associated with significant morbidity and discomfort. In this review, we summarize the evidence from previous clinical studies regarding the optimal surgical margin for skin cancer and the methods for diminishing the complications associated with dermatologic surgery.

**Abstract:**

Despite the significant progress made in the past several years in pharmacotherapies for skin cancer, such as BRAF/MEK inhibitors, immune checkpoint inhibitors, and Hedgehog pathway inhibitors, surgical removal of primary skin cancer is still the first choice of treatment unless distant metastases are evident. In cases of lymph node metastases with clinically palpable lymphadenopathy, lymph node dissection (LND) is typically performed for most skin cancers. In the surgical treatment of primary skin tumors, the surgical margin is critical not only for reducing the possibility of tumor recurrence but also for minimizing the cosmetic and functional complications associated with wide local excision. In contrast, dermatologic surgery can cause various complications. Although skin graft is frequently used for reconstruction of the surgical defect, extensive graft necrosis may develop if optimal stabilization of the graft is not obtained. LND also sometimes causes complications such as intraoperative or postoperative bleeding and postoperative lymphoceles. Moreover, as in other types of surgery, surgical site infection, intraoperative anxiety, and intraoperative and postoperative pain may also develop. These complications are frequently associated with significant morbidity and discomfort. In this review, we summarize the evidence from previous clinical studies regarding the optimal surgical margin for skin cancer and the methods for diminishing the complications associated with dermatologic surgery.

## 1. Introduction

Significant progress in pharmacotherapy for skin cancer has been made in the past several years. Agents targeting aberrant signaling in melanomas with *BRAF* mutations have been shown to significantly improve the survival rate of melanoma patients [1]. In addition, immune checkpoint inhibitors have been developed and approved for the treatment of advanced melanoma and Merkel cell carcinoma [2,3]. Moreover, Hedgehog pathway inhibitors have created a paradigm shift for locally advanced or metastatic basal cell carcinoma (BCC) [4]. However, surgical removal of primary skin cancer is still the first choice of treatment unless distant metastases are evident, although local ablative treatments may be an alternative method when standard surgical excision is not indicated due to significant cosmetic and functional concerns. Lymph node dissection (LND) is also a common method for treatment of most skin cancers in cases of lymph node metastases with clinically palpable lymphadenopathy. In the surgical treatment of a primary skin tumor, the surgical margin is critical since it significantly affects tumor recurrence rate as well as the cosmetic and functional complications associated with wide local excision (WLE). In contrast, dermatologic surgeries including WLE, reconstructive surgery and LND can cause various complications. Although a skin graft is one of the most common methods for reconstruction of the surgical defect when the defect is large and primary closure is difficult, extensive graft necrosis sometimes develops and insufficient stabilization of the graft is a frequent cause of the graft necrosis. Various complications such as intraoperative or postoperative bleeding and postoperative lymphoceles may also be experienced in LND. In addition, although relatively rare in dermatologic surgery, surgical site infection may also develop. Since most of dermatologic surgeries are performed under local anesthesia, a substantial number of patients experience intraoperative anxiety and intraoperative pain, especially in surgeries for facial skin lesions. Moreover, postoperative pain may also occur, and all of these complications are frequently associated with significant morbidity and discomfort. In this review, we summarize the evidence available in the literature on dermatologic surgery, especially focusing on the surgical margin for WLE for each type of skin cancer and on methods that diminish the complications associated with dermatologic surgery.

## 2. Surgical Margin of Primary Tumors

### 2.1. Malignant Melanoma

Melanoma is one of the most aggressive malignant skin tumors and its incidence has been increasing [5]. Malignant melanoma has traditionally been classified into four subtypes according to the clinical and histologic findings: lentigo maligna melanoma (LMM), superficial spreading melanoma (SSM), acral lentiginous melanoma (ALM), and nodular melanoma (NM) [6]. The proportions of the melanoma subtypes differ according to the ethnic population. Whilst rare in white populations, ALM has a much higher incidence in Asian populations: previous studies demonstrated that in white populations, ALM accounts for 1% to 7% of all malignant melanomas, but in Asian populations, for more than 50% [7,8,9]. In the updated World Health Organization classification, melanoma is classified according to the associated cumulative sun damage (CSD), which is related to genetic mutations of tumors [10]. LMM is included in high-CSD melanoma and is typically associated with mutations of *BRAF*^V600K^, *NRAS*, *NF-1,* or *KIT* [11,12]. SSM is included in low-CSD melanoma and frequently harbors *BRAF*^V600E^ mutations [11,12]. Most ALM is included in melanomas not consistently associated with CSD [13], and the common genetic alterations include *KIT* mutations [11,12]. NM is included in high-CSD melanoma, low-CSD melanoma, or melanomas not consistently associated with CSD depending on the location.

WLE is a standard therapy for primary lesions when distant metastases are absent. However, only six randomized clinical trials have been conducted to evaluate the outcomes of melanoma patients with different peripheral surgical margins (Table 1), and the evidence establishing the surgical margin remains insufficient [14]. The peripheral surgical margin suggested in guidelines is typically determined by the tumor thickness, one of the most important factors for the prognosis of melanoma (Table 2) [15,16]. As for melanoma in situ, Bartoli et al. reported that in cases of smaller lesions (less than 2 cm in diameter), no significant difference was found in local recurrence between a 3 mm peripheral margin and a wider margin [17]. In contrast, Kunishige et al. showed that 6 mm and 9 mm margins achieved a complete clearance rate of 86% and 99%, respectively, and they recommended a 9 mm peripheral margin for standard excision [18]. From these results, the current National Comprehensive Cancer Network (NCCN) guidelines recommend a peripheral margin of 5 to 10 mm [19]. A previous study has reported that localization to the head, neck, acral region, genitalia, or pretibial leg and tumor size greater than 1 cm were significantly associated with subclinical spread [20]. Therefore, it may be reasonable that a peripheral surgical margin of between 5 and 10 mm is determined according to the reported risk factors of subclinical spread and associated functional and cosmetic impairments induced by the excision.

Veronesi et al. conducted a randomized control trial evaluating 1 cm versus 3 cm peripheral surgical margins for invasive melanoma with a tumor thickness of ≤2.0 mm and revealed that the relapse-free survival (RFS: defined as the length of time that patients do not show any disease recurrence after treatment), and overall survival (OS: defined as the length of time that patients survive after treatment) were comparable between these groups. These comparable RFS and OS were seen in both lesions with a thickness of <1.0 mm and lesions with a thickness of 1.0 to 2.0 mm [21]. Khayat et al. conducted another randomized control trial evaluating 2 cm versus 5 cm peripheral surgical margins for excision of melanoma with a tumor thickness of <2.1 mm [22]. In their study, the local recurrence rate (LRR: defined as rate of recurrence within 2 cm of the scar after surgery), RFS, and OS were also comparable between the group with lesions of <1.0 mm thickness and the group with lesions of 1.0 to 2.0 mm thickness. In contrast, a retrospective study conducted by Hudson compared 1 cm and 2 cm peripheral surgical margins in melanoma of 1 to 2 mm thickness [23]. In that study, a 1 cm peripheral margin showed higher LRR, although the melanoma-specific survival (MSS) rates, defined as the rate of subjects who have not died from melanoma, were comparable. From these results, the current NCCN guidelines recommend 1 cm and 1 to 2 cm peripheral surgical margins for melanoma with tumor thicknesses of ≤1.0 mm and 1.0 to 2.0 mm, respectively [19]. As for thick melanoma, a randomized controlled trial comparing 2 cm versus 4 cm peripheral surgical margins for melanoma with tumor thickness of 1.0 to 4.0 mm demonstrated no significant difference in the LRR or OS [24]. Similarly, another randomized controlled study comparing 2 cm versus 4 cm peripheral surgical margins for melanoma with tumor thickness of >2.0 mm revealed no significant difference in the LRR, RFS, or OS [25,26]. In contrast, a randomized controlled study conducted by Hayes et al. compared 1 cm versus 3 cm peripheral surgical margins for melanoma with tumor thickness of >2.0 mm and revealed a better MSS rate in the group with 3 cm surgical margins [27]. From these results, the current NCCN guidelines recommend 2 cm peripheral margins for melanoma with thickness of >2 mm [19]. However, the above-described randomized controlled studies to evaluate the peripheral surgical margins comprised mainly white patients, and only a little evidence for appropriate peripheral margins for WLE of ALM has been obtained. A retrospective study focusing only on ALM revealed that local recurrence was also independent of whether the excision margin was 1 cm or more in thin ALM (thickness ≤1 mm) and that a 2 cm margin was associated with a reduced rate of local recurrence when compared with a <2 cm margin in thick ALM (thickness >1 mm) [28]. On the basis of these results, a 1 cm margin and a 2 cm margin may be sufficient for thin ALM and thick ALM, respectively. However, because of the study’s limitations including its retrospective design, further randomized prospective studies with large numbers of patients that evaluate peripheral surgical margins focusing on ALM are needed to establish the optimal margin.

Less focus has been placed on deep surgical margins, and the NCCN guidelines do not make direct recommendations about them [19]. Hunger et al. conducted a retrospective study evaluating the effects of fascia excision on the outcomes of patients with melanoma thicker than 2 mm [30]. In their study, local recurrences, locoregional and distant metastases, disease-free survival, and OS were comparable between the fascia-excised and the fascia-preserved groups. Thus, at least fascial excision is not required for all melanoma lesions, although its requirement is dependent on the degree of tumor infiltration.

Mohs micrographic surgery (MMS), a specialized surgical excision technique, can allow for a smaller initial tumor margin, resulting in healthy tissue conservation [31]. MMS is typically performed by removing a thin margin of tissue circumferentially around and deep to the clinical margins of a skin tumor. It is then rapidly frozen and sectioned. Sectioning is performed in a horizontal direction, allowing virtually 100% of the tissue margin to be evaluated under the microscope. The process is repeated until the tumor has negative histologic margins [31]. MMS provides evaluation of the entire peripheral and deep margins, whereas surgical margins are examined in random vertical sections in typical surgical excision. The disadvantages of MMS include its being cost- and time-consuming [32]. In addition, MMS requires specialized equipment and a trained technician, and therefore, this type of surgery is not universally available [33,34]. Multiple studies suggested the usefulness of MMS for the treatment of melanoma. Zitelli et al. evaluated 369 primary lesions treated with MMS and reported an LRR of 0.5%, which was lower than that (3%) of historical WLE [35]. They also demonstrated lower rates of metastasis after MMS than after historical WLE. Valentin-Nogueras et al. also conducted a retrospective study evaluating MMS for the treatment of 2114 melanoma lesions [36]. They found the LRR with a mean follow-up of 3.73 years to be 0.49%, which was lower than that for the historical WLE group. In addition, Hanson et al. conducted a large-scale database study of 50,397 cases of head and neck melanoma, which comprised 3510 lesions treated with MMS and 46,887 lesions treated with WLE [37], and found that the OS was better in the MMS group after control for potential confounding factors. Another large scale study revealed that MMS was associated with a modest improvement in OS relative to WLE for stage-I tumors classified by the *American Joint Committee on Cancer Staging Manual, 8th edition*, across all sites [38]. However, subgroup analysis revealed that the benefit of MMS was obtained only in head and neck lesions. Consistently, Demer et al. conducted a large-scale database study of melanoma of the trunk and/or extremities and found no significant difference in OS between MMS and WLE [39]. Given these studies and the importance of healthy tissue conservation in the head and neck region, MMS would be useful for the treatment of head and neck melanoma. However, further studies are needed to elucidate the usefulness of MMS for melanoma of other areas. The current NCCN guidelines do not recommend MMS for primary treatment of invasive cutaneous melanoma when standard clinical margins can be obtained [19].

Surgery may also be performed for distant metastases of melanoma. A previous study compared surgery or surgery combined with systemic therapy to systemic therapy alone and showed a better survival in the surgery group [40]. However, this study was conducted in 2012, when checkpoint inhibitors or BRAF/MEK inhibitors were unavailable, and systemic therapy is currently the standard treatment for distant metastases of melanoma. However, several studies suggested that surgery may be beneficial for prognosis even in the era of modern systemic treatment. Puza et al. conducted a retrospective study to evaluate effectiveness of surgery for metastatic lesions after treatment with immune checkpoint inhibitors [41]. In this study, some of the patients showed a good prognosis after surgery, and the authors suggested that select patients with an oligoprogressive disease and a mix response after treatment of immune checkpoint inhibitors may benefit from surgery. In addition, Wankhede recently conducted a meta-analysis to clarify the efficacy of curative metastasectomy (CM) compared with incomplete metastasectomy or nonsurgical treatment [42]. In this study, the authors demonstrated that CM was associated with a lower risk of death compared to non-curative treatment and the outcome was independent of the effective systemic therapy, although selection bias may affect the results of this study. In contrast, surgery might impair the quality of life as well as delay systemic treatment [43]. Therefore, various factors including response to systemic treatment, and size, number and location of metastatic lesions should be carefully considered to conduct surgery for distant metastases of melanoma.

### 2.2. Squamous Cell Carcinoma (SCC)

SCC is characterized by a malignant proliferation of keratinocytes and is the second-most frequent skin cancer after BCC. Whilst most cases of SCC have a mild clinical course, it may show locally aggressive behavior and metastatic potential. The standard treatment for invasive SCC is excision unless distant metastases are evident. Since incomplete excision has been reported to be associated with transition into a poorer degree of differentiation of the residual tumor cells and with poor OS, achieving wide excision with a clear histologic margin is crucial [44,45]. Factors associated with an incomplete surgical margin include tumor depth, tumor size, poor differentiation, head and neck localization, and former incomplete excision [45,46]. In 1992, Brodland et al. reported a retrospective study evaluating the surgical margins and tumor clearance of 141 SCC tumors in MMS [47]. The overall tumor clearance rates were 96% and 99% when the surgical margins were 4 mm and 6 mm, respectively. In tumors with a longest diameter of <1 cm, 100% tumor clearance was achieved with a 4 mm margin. In tumors with a longest diameter of ≥1 cm to <2 cm, the tumor clearance rates were 95% and 100% with surgical margins of 4 mm and 6 mm, respectively. In tumors with a longest diameter of ≥2 cm, the tumor clearance rates were 86% and 97% with resection margins of 4 mm and 6 mm, respectively. The clearance rates of tumors without subcutaneous infiltration were 98% and 100% when the surgical margins were 4 mm and 6 mm, respectively. In contrast, the clearance rates with subcutaneous infiltration were 90% and 98% with surgical margins of 4 mm and 6 mm, respectively. When cases were divided by the histologic grade of differentiation, the clearance rates were 97% and 100% for grade 1, 93% and 97% for grade 2, and 80% and 100% for grade 3/4 when the surgical margins were 4 mm and 6 mm, respectively. They also evaluated the clearance rates for high-risk regions consisting of the scalp, ear, eyelid, nose, lip, and other regions. In the high-risk regions, the clearance rates were 91% and 98% when the resection margins were 4 mm and 6 mm, respectively. In contrast, the clearance rates in the other regions were 98% and 100% with the surgical margins of 4 mm and 6 mm, respectively. From the results, the authors suggested that a surgical margin of at least 4 mm is required and a surgical margin of 6 mm is recommended for SCC with a longest diameter of ≥2 cm, with intermediate or poor histologic differentiation, with development in high-risk regions, or with subcutaneous infiltration in order to achieve a 95% complete excision rate with a histologically clear margin. On the basis of this study and other retrospective studies, the United Kingdom National Multidisciplinary Guidelines define high-risk SCC as having the following features: size >2 cm, failure of previous treatment, immunosuppression, depth or invasion of >2 mm thickness, Clark level >4, perineural invasion, primary site ear or hair-bearing lip, and a poorly differentiated or undifferentiated tumor, and recommend that the surgical margins be ≥4 mm or ≥6 mm in low-risk and high-risk SCC, respectively [48]. The British Association of Dermatologists (BAD) recommends ≥6 mm surgical margins for high-risk SCC and ≥10 mm margins for very high-risk SCC as defined by Keohane et al. [49]. However, wide excision with surgical margins of greater than 6 mm in high-risk regions such as the ear and lip may cause significant cosmetic and functional complications. In addition, in the study conducted by Brodland et al., 100% tumor clearance was achieved with 4 mm surgical margins even in tumors located in high-risk regions when the tumor size was less than 1 cm [47]. Thus, consideration of multiple risk factors and surgical complications might be required to determine the peripheral surgical margin, especially in tumors of the high-risk head and neck region. The NCCN guidelines also define low- and high-risk SCC according to several factors including the location and size and recommend 4 to 6 mm peripheral margins for low-risk SCC, whereas they do not recommend a defined margin for high-risk SCC because of variable clinical characteristics [50].

Thiem et al. evaluated the impact of microscopically clear margin diameters, which was defined as a distance between excision edge and tumor cells evaluated under microscope [51]. They found that a margin size of >4.1 mm for both peripheral and vertical microscopic margins was a negative cutoff value without local recurrence despite no significant association between microscopically clear margin diameters and local recurrence, indicating that a microscopic margin of >4.1 mm is a safe microscopic margin for preventing local recurrence. Phillips et al. also evaluated the histologic margin [52] and found that the 5-year disease-specific survival was better in patients with a pathologic margin distance of ≥5 mm than in those with a margin distance of <5 mm. However, the RFS was comparable, and it may be difficult to explain why only disease-specific survival, not RFS, differed according to the margin distance. One possible explanation is that other confounding factors influencing the histologic margins may also affect the disease-specific survival. Therefore, further studies on the association of the histologic margin with prognostic factors including recurrence and disease-specific survival are needed.

MMS is also effective in the treatment of SCC. Pugliano-Mauro et al. evaluated the outcome of SCC patients treated with MMC and demonstrated that the LRR was only 1.2%, although 4.6% of the tumors involved named nerve trunks [53]. Leibovitch et al. also demonstrated that the LRR of patients who completed the 5-year follow-up after MMS was 3.9% [54]. In their study, the LRR was 2.6% in patients with primary SCC and 5.9% in patients with previously recurrent SCC [54]. Xiong et al. conducted a retrospective study evaluating the outcomes of patients with T2a SCC who were treated with WLE or MMS [55]. They found that the LRR was significantly higher in those treated with WLE (4.0%) than in those treated with MMS (1.2%). Multiple logistic regression analysis revealed that an immunocompromised state and WLE were significantly associated with local recurrence and that WLE, the high-risk head and neck location, and poor histologic differentiation were significantly associated with poor outcomes evaluated by either overall recurrence or disease-specific death. The current NCCN guidelines also recommend MMS as the preferred surgical technique for high-risk SCC [50,56]. Since MMS may not only provide a low LRR but also spare the healthy tissue, MMS is thought to be especially useful for high-risk SCC of the head and neck region.

### 2.3. Basal Cell Carcinoma (BCC)

BCC is the most common malignant tumor of the skin. Histologically, BCC is divided into nodular, superficial, infiltrative, micronodular, morphoeic, and mixed types [57]. Although the majority of BCCs do not develop metastases, BCC lesions sometimes show locally aggressive features [58]. The standard treatment for BCC lesions is surgery, and complete histologic resection is crucial to prevent local recurrence. Local ablative treatments are an alternative method when standard WLE is not indicated due to significant cosmetic and functional concerns, especially in head and neck lesions. Clover et al. conducted a prospective randomized control study evaluating electrochemotherapy (ECT) against standard WLE, and showed comparable LRR between these groups [59]. In addition, cases of BCC successfully treated with radio frequency ablation and carbon dioxide laser ablation have also been reported [60,61]. However, it may be difficult to perform appropriate histological evaluation in lesions treated with local ablation. In addition, Jung et al. revealed that recurrent BCC following ablative laser treatments showed a more aggressive histological type and required more stages of excision in MMS than primary BCC [62]. Therefore, careful follow-up is needed when ablation treatment is selected.

The optimal surgical margin for WLE of BCC is yet to be well established. Kundnani et al. conducted a retrospective study evaluating 120 BCC lesions [58]. They found that local nonaggressive BCC lesions were excised with peripheral margins of 5 mm and of up to 1 cm for the aggressive morphoeic type and that 23 lesions (19.16%) had positive surgical margins. A meta-analysis conducted by Gulleth et al. revealed that the rates of obtaining a pathologically clear margin using resection margins of 2 mm, 3 mm, 4 mm, and 5 mm were 82%, 85%, 85%, and 85%, respectively [63]. Both these studies suggest that a peripheral surgical margin of 5 mm may not be sufficient to achieve complete resection in more than 95% of all BCC lesions [58,63]. Previous reports demonstrated that factors associated with an increased LRR include large tumor diameter, head/neck location, recurrent tumor, poorly defined border, immunosuppression, aggressive histologic growth pattern (such as micronodular, infiltrative, and morphoeic types), and perineural invasion [64,65,66,67,68,69,70]. Zitelli et al. evaluated 117 cases of patients with previously untreated, well-demarcated BCC who were treated with MMS and found that a minimum peripheral margin of 4 mm was necessary to totally eradicate the tumor in more than 95% of the cases [71]. Thus, the current NCCN guidelines define BCC lesions with the following features as low-risk BCC: lesions <2 cm in the trunk or extremities, well-defined lesions, primary lesions, absence of immunosuppression, absence of prior irradiation, nodular or superficial histologic, subtype and absence of perineural involvement, and they recommend a 4 mm clinical peripheral surgical margin for low-risk BCC [72]. A wider margin is recommended for high-risk BCC, but the NCCN guidelines do not specify a defined surgical margin. The BAD guidelines recommend a 4 to 5 mm peripheral margin for low-risk BCC and a greater than 5 mm peripheral margin for high-risk BCC [73]. However, such recommendations were based on studies comprising mainly white patients, and a 4 mm margin is sometimes not feasible owing to functional and cosmetic complications after resection, especially for lesions of the head and neck [71]. Whereas the BCC lesions of white patients are frequently nonpigmented and with unclear clinical borders, those of Asian patients are typically pigmented and well defined [33,74,75,76,77,78]. Therefore, a narrow surgical margin may be sufficient for complete excision of BCC lesions in Asian individuals. Indeed, Ito et al. conducted a retrospective study to evaluate the outcomes of 288 BCC lesions, in which 281 (75.7%) were excised with a narrow margin (≤3 mm), and revealed that only two lesions (0.7%), which were excised with a 2 mm margin, were associated with a positive surgical margin [77]. In their study, narrow-margin excision achieved a complete removal rate of 99% (2 mm margin: 95.3%; 3 mm margin: 100%). Another study analyzing 120 cases of pigmented BCC resected with peripheral margins of 1 to 10 mm that were predetermined at the surgeon’s discretion showed that all low-risk BCC tumors were completely resected within 3 mm, although a greater than 5% incomplete resection rate was seen in high-risk BCC tumors resected with a 3 mm peripheral surgical margin of <3 mm, indicating that a 3 mm peripheral surgical margin might be adequate for low-risk pigmented BCC in Asian patients [78]. Nakamura et al. conducted a retrospective multicenter study of 1000 BCC lesions treated with WLE. In their study, the most common peripheral surgical margin was 3 mm (52%), followed by 2 mm (26%) and 5 mm (11%), and 12 lesions showed a positive surgical margin [34]. The authors evaluated the “accuracy gap” of the clinical tumor border by calculating the difference in the distance between the clinically determined peripheral surgical margin and the histologic tumor side margin, and they also calculated the estimated margin positivity rates (ESMPRs) by the accuracy gap. The median accuracy gap was 0.3 mm, and the ESMPRs with 2 mm and 3 mm peripheral surgical margins were 3.8% and 1.4%, respectively. Multiple logistic regression analyses revealed that the poorly defined lesions showed significantly higher ESMPRs than those of the well-defined lesions for both 2 mm and 3 mm surgical margins. The ESMPRs of well-defined and poorly defined lesions were 3.1% and 11.4%, respectively. Collectively, these results suggest that a surgical margin of 2 mm may be sufficient for WLE of BCC with well-defined lesions in Asian patients.

As compared with peripheral surgical margins, only a few studies have been conducted to analyze deep surgical margins. Takenouchi et al. evaluated the maximum vertical diameter calculated by the distance from the surrounding skin surface to the bottom of the tumor in 235 primary BCC lesions treated with WLE [79]. They found that male sex, larger tumor diameter, and aggressive histologic subtypes including the infiltrative, morpheic, and micronodular types were significantly associated with a larger vertical diameter. Terashi et al. evaluated the presence of muscle invasion by the BCC in each part of the nose (nasal ala, supra-alar crease, lateral nasal dorsum, nasal tip, nasal dorsum and columella, and underside of the nasal tip) [80]. The authors found that BCC of the supra-alar crease had the highest frequency of muscle invasion (86.9%), and they proposed a two-step procedure in this area: first, removal of the BCC lesion including parts of the underlying muscle, and second, reconstruction after confirmation of complete histologic resection. Recently, Matsushita et al. conducted a retrospective multicenter analysis of 718 BCC lesions treated with WLE [81]. In this study, the authors evaluated both the tumor thickness and the tumor invasion level. Tumor thickness was defined as distance from the top of the granular layer to the deepest extension of the tumor; in ulcerated lesion, measurement was from the ulcer base overlying the deepest point of invasion. Tumor invasion level was defined as the deepest part of the tumor cells. In multiple regression analysis, only larger tumor diameter was significantly associated with greater tumor thickness. Larger tumor diameter and anatomic regions (orbit > nose > others) were significantly correlated with tumor invasion level up to the subcutis or to the muscle and below. Incomplete excision at the vertical edge was seen in 1.9% of cases, and the histologic aggressive type (micronodular, infiltrative, morphoeic, and mixed types) showed a higher positive rate than did the nodular type. Larger tumor diameter was also correlated with a positive vertical margin. Collectively, these studies indicate that special attention should be paid to deep margins and that consideration of resection including the muscle layer might be needed especially in large tumors, lesions in the orbital or nasal region (especially the region of the supra-alar crease), and histologic aggressive types to avoid positive deep surgical margins.

As for MMS in BCC, a prospective randomized controlled study comparing WLE versus MMS for primary and recurrent BCC revealed that the LRR of patients treated with MMS for recurrent BCC was significantly lower than that of those treated with WLE [82,83]. Tissue sparing has also been reported to be superior in MMS when compared with that in WLE in the treatment of small nodular BCC [84]. These studies suggest that MMS is also effective for BCC treatment, especially for recurrent BCC. The current NCCN guidelines also recommend MMS as a preferred technique for the treatment of high-risk BCC [72].

### 2.4. Merkel Cell Carcinoma (MCC)

MCC is a rare and aggressive cutaneous neoplasm of neuroendocrine origin. MCC frequently develops in the head and neck region of older adults and is known to be associated with immunosuppression and polyomavirus infection [85,86]. MCC has a high risk of local recurrence after surgical excision even with a wide surgical margin [87]. Since histologic margin positivity is associated with poor OS, complete excision of the primary tumor is important [88,89]. However, evidence supporting a specific margin-size recommendation for WLE of MCC remains insufficient. Perez et al. compared the results in patients with surgical margins of 1 cm, 1.1 to 1.9 cm, and 2 cm or more and showed that the LRR and 5-year disease-specific survival were comparable among the three groups [90]. However, patients with surgical margins of 2 cm or more had the highest rate of requiring reconstruction with a skin flap or graft [90]. Jaouen et al. also compared the results of patients treated with WLE with narrow surgical margins (0.5–1.0 cm) and of those treated with WLE with wide margins (>1.0 cm) and revealed comparable OS and local RFS between the two groups [89]. However, there have also been studies showing conflicting results. Andruska et al. compared the results of MCC patients treated with WLE with margins of <1 cm, 1 to 1.9 cm, and ≥2 cm and demonstrated that every 1 cm increase in the WLE margin was associated with improved local RFS, disease-free survival, and disease-specific free survival [91]. Because MCC is a radiosensitive tumor and adjuvant radiation therapy was shown to improve local control, adjuvant radiation therapy after excision of the primary tumor is frequently applied [92,93,94]. The retrospective studies showing the conflicting results described above were of patients with different rates of adjuvant radiation therapy, which may have contributed to the different effects of the surgical margin length on the LRR and disease-specific survival rate [89,90,91]. Tarabadkar et al. compared the results of MCC patients treated with wider surgical margins (>1 cm) with the results of MCC patients treated with narrower margins (≤1 cm) after dividing the patients on the basis of the absence or presence of adjuvant radiation therapy. In patients receiving WLE alone, patients treated with wider surgical margins developed lower LRR than that of patients treated with narrower margins. However, in patients receiving WLE plus adjuvant radiation therapy, the LRR was low in both groups and did not differ significantly between the two groups [95]. These studies suggest that consideration of the surgical margin might be required depending on the possible subsequent adjuvant radiation therapy. In addition, since the previous studies evaluating the effect of the peripheral surgical margin were retrospective studies, further studies with prospective randomized control designs are required to determine the appropriate peripheral surgical margin in WLE for MCC treatment. Although less focus has been given to deep surgical margins, Wikerson et al. and Jaouen et al. demonstrated that positive histologic margins were more frequently located on the deep sections than on the peripheral sections [88,89]. Deep surgical margins would be dependent on the layer of the estimated tumor infiltration, and it is difficult to determine the deep margin uniformly. Therefore, appropriate evaluation of the layer with tumor infiltration using imaging tests such as ultrasonography and magnetic resonance imaging (MRI) and wide excision with adequate margins from the estimated tumor infiltration would be important. The current NCCN guidelines recommend a 1 to 2 cm peripheral surgical margin to the investing muscle fascia when clinically feasible and with consideration of possible morbidity [73,96]. A high risk of tumor recurrence is associated with chronic immune suppression, presence of HIV infection, past histology of lymphoproliferative malignancies or solid organ transplant, head/neck primary site, large tumors of longer than 1 cm in diameter, and presence of lymphovascular invasion. When one of these risk factors is present, the NCCN guidelines also recommend adjuvant radiation [73,96].

MMS is also thought to be useful for excision of MCC because of its ability to spare the healthy tissue and assess all parts of the margin. Recently, a meta-analysis of studies on MMS for MCC treatment was conducted by Carrasquillo et al. [97]. In that study, the subgroup analysis of stage I MCC revealed that the LRRs for WLE and MMS were similar, suggesting that MMS is a reasonable method in cases in which tissue sparing is important such as those with development of MCC in the head and neck region. The current NCCN guidelines also recommend MMS as an alternative method when it does not interfere with sentinel lymph node biopsy [73,96].

### 2.5. Extramammary Paget Disease (EMPD)

Extramammary Paget disease (EMPD) is a rare neoplasm that usually develops in the apocrine gland-bearing areas of older people [98,99,100]. Most EMPD cases are diagnosed as carcinoma in situ, which usually shows indolent disease progression. However, once Paget cells invade deeply into the dermis, regional lymph node (LN) metastases and distant metastases frequently develop. Individuals with distant metastases have a poor prognosis because of the limited efficacy of chemotherapies [101]. Therefore, early detection and appropriate treatments before the development of metastases is vital. As in most other skin cancers, WLE is the standard treatment for EMPD unless distant metastases are evident. However, the standard peripheral surgical margin has yet to be well established. Most previous reports had recommended a 2 to 5 cm margin of healthy skin for WLE because EMPD is sometimes multifocal and presents a discontinuous subclinical extension [102,103,104]. However, a previous retrospective study analyzing EMPD patients surgically treated with a 1 cm peripheral margin found that the clinically determined border of well-defined lesions of EMPD corresponded well to the histopathologic border, with a small, microscopic gap between the histopathologic and clinical borders [105]. In another study, conducted by Hatta et al., 5 of 66 patients who underwent curative surgical excision developed local recurrence, but the peripheral surgical margin (≤2 cm or >2 cm) did not correlate with the local recurrence [106]. These studies suggest that a 1 to 2 cm margin is adequate for the peripheral surgical margin in WLE for EMPD treatment. As EMPD sometimes has an ill-defined border or extends beyond the clinical borders, mapping biopsy has been frequently used to reduce the rate of positive surgical margins [107]. Kaku-Ito et al. performed a retrospective study of 133 patients with 150 primary EMPD lesions to evaluate the usefulness of mapping biopsy [108]. The results showed that only 1.6% of the mapping biopsy specimens from well-defined EMPD were positive. Moreover, 4.6% of the mapping biopsy specimens from ill-defined EMPD were positive, whereas all the specimens taken from sites of 2 cm or more from the clinical border were negative. From these results, they suggested that mapping biopsy is not required for well-defined EMPD or when a 2 cm margin can be achieved. They also recommended the peripheral surgical margins to be 1 cm and 2 cm for well-defined EMPD and ill-defined EMPD, respectively. However, there may be cases in which WLE with a 1 cm margin presents difficulty owing to significant functional or cosmetic complications, especially those with resection of the urethra or anus, and in such cases, consideration for limiting the extent of resection might be needed. No studies have been conducted focusing on deep surgical margins in EMPD, and the need for a deep margin is thought to depend on the extent of vertical infiltration of the tumor cells. However, because epidermal Paget cells extend into appendages such as hair follicles and sweat glands, excision should include the appendages even in in situ EMPD.

MMS may also be useful for management of EMPD [102]. However, because EMPD typically presents as a large skin lesion, MMS may be much more expensive and time-consuming than WLE for such large lesions. To shorten the time taken for the MMS procedure, peripheral MMS, in which the periphery of the tumor is marked and excised until a clear margin is achieved, and its modified method have been developed [109,110]. Using this method, MMS in EMPD may become more common and useful in the future.

### 2.6. Dermatofibrosarcoma Protuberans (DFSP)

DFSP is a rare, low-grade soft-tissue sarcoma. Fewer than 5% of patients with DFSP develop distant metastases [111]. Although DFSP occurs predominantly in the trunk and proximal extremities, it may develop throughout the body, including the head and neck region [112,113,114,115,116]. Genetically, *COL1A1–PDGFB* fusion is detected [117]. The mainstay of treatment is complete surgical excision unless metastases are present [118]. However, owing to local infiltrative growth and subclinical extension, incomplete excision is relatively common and positive or close margins are reported to be predictive factors for local recurrence [114,119,120]. Of all the regions, the head and neck region, in which wide margins are difficult to achieve, is known to be significantly associated with recurrence [119,120]. However, consensus regarding the peripheral surgical margin for DFSP has yet to be fully established. WLE with a margin of more than 3 cm has long been recommended for DFSP treatment [121,122,123]. A previous meta-analysis revealed that WLE with a 4 cm surgical margin provides 95% tumor clearance [124]. Another meta-analysis of eight observational studies revealed a significant positive correlation between a margin of more than 3 cm and the LRR and positive surgical margin rate [125]. However, the larger the surgical margin is, the more significant the cosmetic and functional problems that develop after WLE may be [126]. Recently, WLE with a lesser peripheral surgical margin has also been reported to be associated with a low LRR and a low positive surgical margin rate [127,128]. Farma et al. demonstrated a 97% clear histologic margin and a 1% LRR for wide local excision with a 2 cm clinical margin [127]. In contrast, Huis In’t Veld et al. investigated pathologic clear margins and demonstrated no significant influence of the width of the pathologic clear margin on the LRR if the pathologic clear margin was longer than 1 mm [126], suggesting that a 1 mm pathologic clear margin might be sufficient and that additional resections or adjuvant radiotherapy might not be needed, although further studies are required to substantiate this conclusion. The authors also demonstrated that the median difference between the primary surgical macroscopic and the pathologic margin was 22 mm. From these previous studies, a clinical surgical margin of at least a 2 to 3 cm would be needed to achieve complete excision in most cases. The current NCCN guidelines recommend a 2 to 4 cm peripheral margin in WLE [73,129]. No studies have been conducted extensively focusing on deep surgical margins for DFSP treatment. As DFSP usually develops from the superficial subcutaneous layer, the deep fascial layer as a barrier tissue should be included in the excised tissues. In cases with tumor infiltration into the deep subcutaneous layer, further excision including the muscle layers may be needed, depending on the tumor’s location. The current NCCN guidelines recommend that deep margins extend to the investing fascia of the muscle [73,129].

Multiple meta-analyses and retrospective studies have also investigated the usefulness of MMS for the excision of DFSP and demonstrated a lower LRR with MMS than with WLE, although no randomized controlled trials have been conducted to compare MMS and WLE for DFSP treatment [118,119,130,131,132,133]. MMS can not only decrease the LRR but also minimize the surgical margin and preserve the healthy tissues, resulting in the minimization of cosmetic and functional issues after tumor excision [118]. Serra-Guillen analyzed 222 cases of DFSP treated with MMS and showed that the mean minimum peripheral margin for achieving tumor clearance was 1.23 cm [134]. Therefore, although MMS requires multiple excisions and takes more time than WLE, MMS should be considered for DFSP treatment, especially for head and neck DFSP, which shows a higher LRR and severer cosmetic and functional issues after excision than do other regions. The current NCCN guidelines recommend MMS rather than WLE if MMS is available [73,129].

### 2.7. Atypical Fibroxanthoma (AFX)

AFX is a rare, low-grade superficial sarcoma with metastatic potential that frequently presents as a red nodule or plaque. It typically develops on the head and neck of sun-damaged older people. Jibbe et al. conducted comprehensive research addressing excisional surgery of AFX in terms of the surgical margin [135]. They identified 101 AFX lesions in 99 patients and showed that the peripheral clearance margin calculated to clear 95% of all tumors was 2 cm. They also suggested that AFX of 1 cm or less required a peripheral margin of 1 cm. However, since this study was mainly composed of results from case reports and series, it has a potential publication bias. Because AFX frequently develops in the face and neck, wide resection with a large margin may cause significant cosmetic problems. Therefore, a surgical margin of less than 1 cm might be appropriate when the clinical margin of the AFX lesion is clear, although further studies are needed. Jibbe et al. also showed that the overall LRR for primary AFX lesions treated with MMS was 8.3%, and for those treated with WLE, 26.7%. In addition, MMS can allow for smaller initial tumor margins. Thus, MMS should also be considered for AFX treatments to reduce the LRR as well as postoperative complications if MMS is available.

### 2.8. MMS

MMS is effective and should be considered for most skin cancers as described above. Previous studies evaluating the effect of MMS and the recommendations of the current NCCN guidelines for each skin cancer are summarized in Table 3.

## 3. Method for Skin Grafts

Skin grafts are one of the most common methods for the reconstruction of large skin defects after wide excision of skin cancer. Skin grafts are generally classified into split-thickness skin grafts (STSG) or full-thickness skin grafts (FTSG). FTSG is usually harvested from the groin, chest, and upper arm, and the donor site defect is closed by primary closure. STSG is generally harvested with a dermatome, and the donor site area is left for re-epithelialization. Therefore, STSG has a larger donor site availability. The amount of dermis in the graft is associated with higher graft survival and lower degree of contracture. Therefore, STSG has better survivability but shows severer contracture than does FTSG. In contrast, FTSG shows better cosmetic and functional results with less contraction. However, FTSG needs a substantial vascularity to survive. To improve the vascularity, a method of FTSG with a preserved subcutaneous vascular network has been tried [136,137]. This method is also considered to help maintenance of the elasticity and texture of the skin graft [136,137,138]. However, since comparative studies analyzing results showing the presence and absence of a preserved subcutaneous vascular network have not been reported, the efficacy of this method is still unclear and further studies are required to determine the efficacy.

For successful reconstruction of both STSG and FTSG, optimal stabilization of the graft is needed. The tie-over technique is frequently used for the stabilization of skin grafts; Vaseline gauze, cotton pads, or polyurethane sheets are also used [139]. However, significant graft loss may occur when the graft is not fixed to the defect for an adequate period of time. The common causes of graft detachment include the formation of seroma or hematoma under the graft and the shear forces of the interface [140]. Reported risk factors for increased graft loss due to uneven pressure being applied to the skin graft include defects of a large size, exposure of the muscle, and location in the trunk or extremities [141,142,143]. In contrast, negative pressure closure (NPC) has been reported to reduce wound edema, improve bacterial clearance from the wound, and increase local perfusion and was initially used to promote wound healing [144]. However, recent studies have also shown that NPC may be useful for the stabilization of skin grafts as it provides a uniform pressure to affix the graft to the defect [145]. Randomized trials comparing NPC and dressing methods consisting of Vaseline gauze, cotton pads, or polyurethane sheets have shown that NPC is superior to other conventional methods for the stabilization of skin grafts [141,144,146]. Llanos et al. conducted a randomized controlled trial comparing the results of STSG in an NPC group and a control group with use of a polyurethane sheet only and showed lower graft loss of the STSG and shorter hospital stay in the NPC group than in the control group [143]. Petkar et al. also conducted a randomized control study comparing the results of NPC with those of conventional dressing consisting of Vaseline gauze and cotton pads and revealed that the graft survival rate of NPC was significantly higher than that of conventional dressing [145]. These studies suggest that NPC is superior to several conventional methods. However, because no comparative studies had been conducted demonstrating NPC’s superiority over the tie-over technique for graft survival, we previously conducted a retrospective study to compare NPC and the conventional tie-over method for stabilization of STSG using data of patients with defects in the trunk or extremities of >10 cm or with muscle exposure [146]. We found that NPC achieved a significantly higher skin graft survival rate than that of the tie-over method. In addition, NPC showed a tendency toward a shorter time from the harvesting of the skin graft to the end of the NPC graft stabilization than that of the tie-over method. Then, we also conducted a prospective, phase-II clinical study to investigate the safety and efficacy of NPC for the stabilization of STSG in large (longest diameter, >10 cm) or muscle-exposing defects [147]. In that study, the mean survival rate of the skin graft was higher than 95% at day 10, and the mean time from harvesting of the skin graft to the end of NPC graft stabilization was shorter than 20 min, suggesting that NPC for stabilization of STSG leads not only to a high survival rate of the skin graft but also to a short operative time. In addition, no adverse events associated with NPC were observed in the seven enrolled patients, indicating that NPC for graft stabilization is a safe and feasible method. Collectively, our studies’ findings suggest that NPC for stabilization of STSG in large or muscle-exposing defects is superior to the conventional tie-over method. Although NPC achieves a higher survival rate of the graft and a shorter operative time, given its expense [148], it may not be cost-effective to apply for stabilization of all STSGs. Therefore, we suggest that NPC may be most useful for graft stabilization for large or muscle-exposing defects. However, since NPC has a potential to promote tumor growth, caution is warranted and further prospective studies for evaluating tumor recurrence with larger number of patients and long-term observation after use of NPC are also needed [149,150,151].

As for the donor site of STSG, the thighs are predominantly used. However, several studies demonstrated that the occipital donor site provides good results in terms of graft survival and healing rates [152,153,154]. Kovacs et al. conducted a randomized controlled study to compare the results of STSG by donor site after tumor resection [155]. The study participants were randomized to undergo STSG from the occipital region or the thigh, and the occipital donor sites showed significantly faster healing, less pain, fewer complications, and a better cosmetic outcome. In addition, the occipital donor sites provided better graft survival rates, indicating that the occipital region is superior to the thighs as a graft donor site. The good result of the occipital donor site may be due to the good vascularization of the occipital region [152]. Another explanation is that the perifollicular portions of the hair shafts contain an epidermal stem cell pool, which would promote re-epithelialization [156,157,158]. However, in that study, alopecia at the occipital donor site occurred in two patients. Previous studies also showed that more than 4% patients had microalopecia at the occipital donor site [153,159]. Iatrogenic alopecia may significantly decrease patient satisfaction. Therefore, surgeons should be careful not to harvest occipital region grafts too deeply. Since the frequency of complications in the donor site would be significantly associated with the surgeon’s technique, further multicenter studies with different surgeons are desired to determine the usefulness of this method.

## 4. Methods to Diminish Complications Associated with LND

LND is sometimes associated with various complications such as intraoperative or postoperative bleeding, postoperative pain, and lymphoceles, and lymphoceles are known to be one of the main causes of seroma formation [160,161]. These complications are frequently associated with significant morbidity and discomfort, and methods or techniques for diminishing the complication are therefore required. The Harmonic Scalpel (HS) is a system allowing cutting and hemostasis without the application of electrical energy [162]. The HS has been shown to reduce complications and operative times in both open and laparoscopic surgeries [163,164,165]. The HS is based on mechanical energy produced by ultrasound, and such mechanical energy leads to tissue denaturation and generation of coagulum without high thermal injury [162]. Previous studies evaluated the usefulness of the HS in axillary LND, but the results differed between each study. Lumachi et al. conducted a case-control study and demonstrated a significant decrease in drainage volume after axillary LND in breast cancer patients treated with HS surgery [166]. Similarly, a randomized controlled study conducted by Iovino et al. revealed that use of the HS for axillary LND decreased the drainage volume, seroma formation, intraoperative bleeding, and hospitalization stay, suggesting that the HS produces an effective sealing of the lymph and blood vessels [167]. They also showed a lower demand for painkiller medications in patients with use of the HS, and this lower demand might be explained by the lower thermal effect on tissues and less inflammatory reaction induced by the HS. They suggested that cutting all of the tissues only with the HS is important because it may ensure the perfect sealing of lymphatic and blood vessels and cause lesser thermal injury and inflammation. A randomized study conducted by He et al. also demonstrated that using the HS significantly diminished the operative time, bleeding, total drainage volume, hospitalization stay, and visual analog scale of postoperative pain as compared with traditional electrocautery, indicating that axillary LND using the HS is a feasible and useful method [168]. However, several subsequent studies failed to show better results including those related to drainage volume and rate of seroma development by use of the HS [169,170]. In a randomized study conducted by Gie et al. evaluating the usefulness of the HS for inguinal LND and axillary LND in patients with skin cancer or sarcoma, the HS did not influence the amount of drainage after LND [170]. Therefore, the effect of HS use in axillary and inguinal LND might be significantly dependent on the surgeons’ techniques, and further multicenter studies are required to determine its usefulness.

Multiple studies also evaluated the usefulness of the HS for neck LND, and most suggested positive results for its usefulness. Verma et al. conducted a randomized study of 40 patients who were to undergo selective neck LND for primary oral malignancies and showed that use of the HS was significantly associated with a smaller amount of intraoperative bleeding [171]. In addition, a recent a meta-analysis of seven randomized control trials demonstrated that the HS group had a significantly shorter operative time and a lower amount of intraoperative bleeding and of drainage fluid volume [172]. These studies suggest the usefulness of HS for diminishing the complications associated with neck LND. However, as mentioned above, the effect of the HS may be dependent on the surgeon’s technique, and further multicenter studies with multiple different surgeons are desired to elucidate whether its usefulness for neck LND is universal.

Several studies evaluated the effects of dyes for detection of lymphatic leakage to prevent the development of lymphoceles. Nakamura et al. evaluated the effect of isosulfan blue injection around the dissected inguinal region to identify and ligate leaked lymphatic vessels in inguinal LND and revealed that the postoperative drainage output and duration in this method were significantly lower and shorter than those of conventional inguinal LND, respectively [173]. Ravisankar et al. conducted a randomized clinical trial evaluating the potential role of intraoperative mapping of lymphatic leakage with methylene blue injection and of clipping the leaked lymphatic vessels in inguinal LND and found that the drainage output and duration in the interventional method with use of methylene blue injection were also significantly lower and shorter than those of conventional inguinal LND, respectively [174]. They reported that only 10 min was needed to conduct the methylene blue injection and clipping of the leaked lymphatic vessels before the closure of the wound. These studies indicate that injection of dyes, including isosulfan blue and methylene blue, to detect and ligate the leaked lymphatic vessels is a useful method to diminish the development of lymphoceles. However, both isosulfan blue and methylene blue are known to induce several adverse events such as allergic reactions, which might be associated with the injection site [151,175]. Therefore, further prospective studies with larger numbers of patients are desired to elucidate the safety as well as the effectiveness of the method.

## 5. Surgical Site Infection (SSI) after Excision of Skin Cancer

SSI is one of the major postoperative complications of surgery. Although the rate of SSI in dermatologic surgeries is low, it may cause significant morbidity such as ruptured sutures and skin graft necrosis [176]. Previous studies have suggested an association of various clinical factors with SSI development following dermatologic surgery, although no consensus has been established. Liu et al. reported that larger defects, those located at the ear, closure with flaps, and secondary intention were independent risk factors for SSI [176]. Kulichova et al. showed that the head and neck region, lips and oral mucosa, acral region, older age, worse preoperative state of the surgical site, and surgeons with less experience were factors associated with a high incidence of SSI [177]. Dixon et al. conducted a prospective observational study of 2424 patients and demonstrated that the lips, ears, perineum, inguinal area, and regions below the knee were associated with a higher risk of SSI [178]. Although infection rates by body location vary depending on each report, the lips, ears, inguinal area, and lower extremities have been frequently associated with higher risk of SSI, and clinicians should be more careful for SSI in dermatologic surgeries of such locations [176,177,178,179,180,181]. It has also been reported that the incidence of SSI is more frequent in tropical regions such as Brazil and some African countries [182,183,184]. This may be due to low-income settings, but high temperature and humidity might also be associated with the high incidence of SSI. Indeed, Anthony et al. demonstrated that monthly SSI rates increased with average temperature [185]. Fortaleza et al. also revealed that higher temperature and humidity were associated with higher frequency of SSI [186]. Therefore, risk of SSI also varies depending on the region and season, and clinicians should also take account of these factors.

Less focus has been directed in previous studies to the association of the incidence of SSI with skin tumor type. Therefore, we also conducted a retrospective study to evaluate factors associated with SSI development in outpatient surgery with a more detailed tumor classification [187]. We found that many clinical factors were associated with SSI; however, multivariate analysis revealed that only invasive SCC, Bowen disease (BD), and actinic keratosis (AK) were independent risk factors for SSI development. BD and AK are regarded as in situ SCC. A chi-square test also showed that the frequency of SSI in invasive SCC, BD, and AK was significantly higher than in other non-SCC tumors, with the frequencies being more than eight times higher. Therefore, our results indicate that dermatologic surgeries for excision of SCC have high risk for SSI development and that extensive care such as preoperative intravenous administration of antibiotics and careful disinfection for such surgeries might be needed. However, our study was limited by the relatively small number of participants and its retrospective nature. Therefore, we plan to conduct further large-scale prospective studies in the future.

## 6. Reduction of Anxiety and Pain Associated with Surgery for Skin Cancer

Many dermatologic surgeries for excision of skin cancer are performed under local anesthesia. Therefore, a considerable number of patients can experience significant anxiety and pain during surgery for skin cancer, especially excision of face lesions. Excessive anxiety and pain can cause elevation of blood pressure and heart rate, which may result in increased bleeding [188]. Nonmedication methods such as music and guided imagery are suggested to reduce pain and anxiety about the surgery and are practically used [189,190]. A previous systemic review revealed that listening to music reduced pain and anxiety in surgical patients [191]. In addition, another systemic review from eight studies showed that guided imagery reduced anxiety during surgery [189]. However, the usefulness of music and guided imagery has yet to be well established for surgery for skin cancer. Vachiramon et al. performed a randomized controlled trial to evaluate whether music reduces anxiety during MMS [192]. The study participants were randomly allocated to listen to self-selected music or to receive dermatologic surgery without music. The results showed that the participants who listened to music during surgery had significantly lower anxiety as assessed with the visual analog scale (VAS) and the State-Trait Anxiety Inventory (STAI). Alam et al. also conducted a randomized control study to evaluate the effect of guided imagery and relaxing music for pain and anxiety during excision of BCC or SCC in the face under local anesthesia [193]; they found no significant differences in patients’ pain and anxiety as assessed with the VAS and STAI. In addition, the blood pressure and pulse rate were comparable across the groups. However, interestingly, surgeon anxiety was significantly lower in the guided-imagery and relaxing-music group than in the control group. The authors suggested that lower surgeon anxiety may correlate with improved surgical outcomes such as scarring, aesthetic quality of repair, or risk of adverse events, although this study did not assess such surgical outcomes.

Hand-holding by nurse or stress balls as a distraction could be other methods for reducing anxiety and pain during surgery under local anesthesia. Previous studies in nondermatologic fields demonstrated that hand-holding by nurses can reduce anxiety during local anesthetic procedures [194]. In addition, it has been reported that intraoperative anxiety and pain ratings were lower in the group that used stress balls than in the control group [195]. Yanes et al. also conducted a randomized clinical trial evaluating the effect of stress-ball use or hand-holding on anxiety during skin cancer excision under local anesthesia [196] but found that neither stress-ball use nor hand-holding reduced anxiety. The authors suggested that stress balls and hand-holding may still provide stress relief in patients who are particularly anxious before the procedure or are comforted by human touch.

Antianxiety agents are sometimes used in dermatologic surgery. Among them, midazolam is a short-acting benzodiazepine and relatively safe medication [197]. Ravitskiy et al. performed randomized controlled and prospective studies evaluating the efficacy of oral midazolam in patients who received MMS for BCC or SCC in the head and neck region [198]. In their study, oral midazolam significantly diminished anxiety and alertness in both the randomized and the prospective arms. In addition, oral midazolam was not associated with major intraoperative or postoperative complications. Therefore, oral midazolam is an effective and safe method to reduce anxiety related to dermatologic surgery.

To reduce postoperative pain, analgesics are frequently used. However, which medications are effective is not yet well known. Sniezek et al. conducted a randomized, double-blind, controlled study comparing the efficacy of analgesics after MMS and reconstruction for head and neck skin cancer [199]. In their study, the patients received 1000 mg acetaminophen, 1000 mg acetaminophen plus 400 mg ibuprofen, or 325 mg acetaminophen plus 30 mg codein immediately after the surgery and every 4 h up to four doses. As a result, the combination treatment of acetaminophen plus ibuprofen showed the lowest pain scores at each postoperative record time interval. In addition, the combination treatment of acetaminophen plus ibuprofen revealed a significantly smaller change from the baseline pain scores than those of the other groups. Complications in the combination treatment of acetaminophen plus ibuprofen were significantly lower than in the combination treatment of acetaminophen plus codein and comparable to those of acetaminophen monotherapy. These results indicated that the combination treatment of acetaminophen plus ibuprofen is an effective and safe method to control postoperative pain after skin cancer excision and reconstruction.

## 7. Conclusions

In this review, we have summarized the evidence from previous studies related to surgical margin for skin cancer and methods for diminishing complications associated with dermatologic surgeries. There have been multiple studies on the surgical margins for skin cancer, and guidelines recommend the surgical margin size in some skin cancers on the basis of those studies. However, skin cancer in each patient has different characteristics such as tumor location, histologic subtype, tumor diameter, and tumor thickness. Therefore, such characteristics should be carefully considered to determine the surgical margin. In addition, the optimal surgical margin in a skin cancer may also differ according to the population. Several studies have indicated that narrower margins might be adequate for BCC lesions in Asians as compared with those in white individuals, as described above [30,65,66]. Therefore, the ethnic population should also be considered to determine the surgical margin.

Although there have also been multiple studies on methods for diminishing complications associated with dermatologic surgery, these studies may have some limitations such as low numbers of participants and retrospective designs. In addition, most studies for evaluating surgical methods may be dependent on specific surgeons’ techniques. Therefore, further high-quality studies including randomized trials with large numbers of patients and multicenter studies with multiple surgeons are required to further develop and establish methods to diminish complications in dermatologic surgery.

We hope that this review provides an up-to-date overview of the optimal surgical margin for skin cancer and methods for diminishing complications associated with dermatologic surgery and that it facilitates the determination of methods in clinical practice.

## Figures and Tables

**Table 1:** Previous randomized controlled trials to evaluate peripheral surgical margins for melanoma.

Year	Researchers	Number	Thickness, mm	Location	Peripheral Margin, cm	Recurrence	OS Rate and/or MSS Rate	Follow-Up Period
1991	Veronesi et al. [21]	612	≤2	Except lesions of face, finger or toes	1 vs. 3	8 year-RFS rate: 81.6% in the 1 cm margin vs. 84.4% in the 3 cm margin (*p* = 0.74)	8 year-OS rate: 89.6% in the 1 cm margin vs. 90.3% in the 3 cm margin (*p* = 0.64)	Mean, 91 months in 1 cm margin and 90 months in the 3 cm margin
2001	Balch et al. [24]	468	1–4	Head/neck or extremity or trunk	2 vs. 4	LRR: 2.1% in the 2 cm margin vs. 2.6% in the 4 cm margin (no significant difference)	10 year-MSS rate: 70% in the 2 cm margin vs. 77% in the 4 cm margin (*p* = 0.074)	Median, 10 years
2003	Khayat et al. [22]	326	≤2	Except ALM	2 vs. 5	LRR: 0.6% in 2 cm margin vs. 2.4% in the 5 cm margin (no significant difference)	10 year-OS rate: 87% in 2 cm margin vs. 86% in the 5 cm margin (*p* = 0.56)	Median, 16 years
2012	Hudson et al. [23]	576	1–2	Whole body	1 vs. 2	LRR: 3.6% in 1 cm margin vs. 0.9% in the 2 cm margin (*p* = 0.044)	OS rate was not significantly different **	Median, 38 months
2004 2016	Thomas et al. [29] Hayes et al. [27]	900	>2	Trunk or limbs (excluding palms and soles)	1 vs. 3	Locoregional recurrence * rate up to 3 years was significantly higher in the 1 cm margin (*p* = 0.02)	OS rate was not significantly different (*p* = 0.14). MSS rate was significantly better in the 3 cm margin (*p* = 0.041) ***	Median, 5.7 years
2011 2019	Gillgren et al. [26] Utjes et al. [25]	936	>2	Trunk or upper or lower extremities	2 vs. 4	LRR: 4.3% in 2 cm margin vs. 1.9% in the 4 cm margin (*p* = 0.06)	Neither OS rate nor MSS rate was significantly different (*p* = 0.75 and *p* = 0.61, respectively) ****	Median, 6.7 years for analyses of LRR Median, 19.6 years for analyses of OS rate and MSS rate

LRR: local recurrence rate; RFS: relapse-free survival; OS: overall survival; MSS: melanoma-specific survival; ALM: acral lentiginous melanoma. RFS rate is defined as the rate of subjects who do not show any disease recurrence. OS rate is defined as the rate of subjects who have not died by any causes. MSS rate is defined as the rate of subjects who have not died from melanoma. * Locoregional recurrence includes local recurrence, in transit metastasis and regional lymph node metastasis. ** There were 14 deaths (6.2%) in the 1 cm group and 33 deaths (9.4%) in the 2 cm group. *** Overall, 253 deaths (55.8%) occurred in 453 patients in the 1 cm group and 241 (53.9%) occurred in 447 patients in the 3 cm group (*p* = 0.14). In total, 194 deaths (42.8%) were attributed to melanoma in the 1 cm group, as compared with 165 (36.4%) in the 3 cm group (*p* = 0.041). **** There were 304 deaths (49%) in the 2 cm group and 317 deaths (51%) in the 4 cm group (*p* = 0.75). In total, 192 deaths (48%) were attributed to melanoma in the 2 cm group, as compared with 205 (52%) in the 4 cm group (*p* = 0.61).

**Table 2:** Comparison of guideline-suggested peripheral margin in melanoma [16].

Organization		Peripheral Margin				
	Tumor Thickness (Breslow) in Melanoma	In Situ	<1 mm	1–2 mm	2.01–4 mm	4 mm<
NCCN		0.5–1 cm	1 cm	1–2 cm	2 cm	
AAD		0.5–1 cm	1 cm	1–2 cm	2 cm	
ESMO		0.5 cm	1 cm		2 cm	
CCA			1.01–2 cm			2 cm

NCCN: National Comprehensive Cancer Network. AAD: American Association of Dermatology. ESMO: European Society of Medical Oncology. CCA: Cancer Council Australia.

**Table 3:** Previous representative studies to evaluate the effect of MMS and recommendations of the current NCCN guidelines for MMS for each skin cancer.

Type of Cancer	Year	Researchers	Type of Study	Number	Limitation of Location or Stage	LRR	Prognosis (Survival or Development of Metastases)	Follow-Up Period	Recommendation of NCCN Guidelines
Melanoma	1997	Zitelli et al. [35]	Retrospective study	MMS, 369	None	MMS, 0.5%; historical WLE, 3%	5-year OS rate: 93%	5 years except for 3 patients	The NCCN guidelines do not recommend MMS for primary treatment of invasive cutaneous melanoma when standard clinical margins can be obtained [19].
	2016	Valentin-Nogueras et al. [36]	Retrospective study	MMS, 2114	None	MMS, 0.49%; historical WLE, 9–20%	5-year disease-specific survival rate: 98.5%	Mean, 3.73 years	
	2019	Hanson et al. [37]	Retrospective study	MMS, 46,887; WLE, 3510	Head and neck	NA	A better OS in MMS than in WLE (WLE HR, 1.181; 95% CI, 1.08–1.29; *p* < 0.001)	NA	
	2019	Cheraghlou et al. [38]	Retrospective study	MMS, 3234; WLE, 67,085	Stage I *	NA	A better OS in MMS than in WLE (HR, 1.16; 95% CI, 1.03–1.32)	Mean, 4.81 years	
	2020	Demer et al. [39]	Retrospective study	MMS, 4413; WLE, 184,449	Trunk and extremities	NA	No significant difference in OS (WLE HR, 1.03; 95% CI, 0.94–1.13; *p* = 0.51)	NA	
SCC	2005	Leibovitch et al. [54]	Prospective observational study	381	None	Primary SCC, 2.6%; recurrent SCC, 5.9%	No cases with metastases during the follow-up period	5 years	The NCCN guidelines recommend MMS as the preferred surgical technique for high-risk SCC [50,56].
	2010	Pugliano-Mauro et al. [53]	Retrospective study	260	None	1.2%	Six tumors (2.3%) metastasized.	A minimum of 2 years	
	2019	Xiong et al. [55]	Retrospective study	MMS, 240; WLE, 126	T2a **	MMS, 1.2%; WLE, 4.0% (*p* = 0.03)	A significantly lower cumulative incidence of overall disease progression *** in MMS than in WLE (WLE HR, 2.9; 95% CI, 1.1–7.6; *p* = 0.03)	Mean, 2.8 years	
BCC	2008 2014	Mosterd et al. [83] Van Loo et al. [82]	Prospective randomized controlled study	MMS, 198; WLE, 199 in primary BCC; MMS, 100; WLE, 102 in recurrent BCC	Face	MMS, 4.4%; WLE, 12.2% in primary BCC (*p* = 0.10); MMS, 3.9% vs. WLE, 13.5% in recurrent BCC (*p* = 0.023)	NA	Median, 79.2 months in primary BCC and 85.0 months in recurrent BCC	The current NCCN guidelines recommend MMS as a preferred technique for the treatment of high-risk BCC [72].
MCC	2022	Carrasquillo et al. [97]	Systematic literature search	MMS, 58; WLE, 4216	Stage I *	MMS, 8.5%; WLE, 6.8% (*p* = 0.53)	No significant difference in regional and distant metastases between MMS and WLE	NA	The NCCN guidelines recommend MMS as an alternative method when it does not interfere with sentinel lymph node biopsy [73,96].
EMPD	2004	Hendi et al. [102]	Retrospective study	27	None	MMS, 26%; historical WLE, 33–60%	Two patients developed metastases	Mean, 58.6 months; median, 55.5 months	NA
DFSP	2008	Paradisi et al. [131]	Retrospective study	MMS, 41; WLE, 38	None	MMS, 0%; WLE, 13.2% (*p* = 0.016)	No cases with metastases during the follow-up period	Mean, 5.4 years in MMS and 4.8 years in WLE	The NCCN guidelines recommend MMS rather than WLE if MMS is available [73,129].
	2021	Gonzalez et al. [118]	Retrospective study	41	Head and neck	MMS, 2.4%; historical WLE, 9–73%	No cases with metastases during the follow-up period	Mean, 92.6 months	
	2022	Serra-Guillen et al. [134]	Retrospective study	222	None	0.9%	NA	Mean, 71.5 months; median, 61.5 months	
AFX	2021	Jibbe et al. [135]	Systematic literature search	MMS, 71; WLE, 30	None	8.3% (MMS) vs. 26.7% (WLE)	NA.	Mean, 41.4 months	NA

MMS: Mohs micrographic surgery; NCCN: National Comprehensive Cancer Network; LRR: local recurrence rate; OS: overall survival; WLE: wide local excision; NA: not applicable; HR: hazard ratio; CI: confidence interval; SCC: squamous cell carcinoma; BCC: basal cell carcinoma; MCC: Merkel cell carcinoma; EMPD: extramammary Paget disease; DFSP: dermatofibrosarcoma protuberans; AFX: atypical fibroxanthoma. * Stage-I tumors were classified according to the *American Joint Committee on Cancer Staging Manual, 8th edition.* ** T2a tumors were defined as having 1, but not multiple, high-risk features (tumor diameter ≥2 cm, poorly differentiated histology, perineural invasion ≥0.1 mm, and tumor invasion beyond fat). *** Disease progression was defined as either overall recurrence or tumor-specific death.
